# Biological monitoring and the influence of genetic polymorphism of As3MT and GSTs on distribution of urinary arsenic species in occupational exposure workers

**DOI:** 10.1007/s00420-014-1009-7

**Published:** 2014-12-10

**Authors:** Beata Janasik, Edyta Reszka, Magdalena Stanislawska, Edyta Wieczorek, Wojciech Fendler, Wojciech Wasowicz

**Affiliations:** 1 Department of Toxicology and Carcinogenesis, Nofer Institute of Occupational Medicine, Lodz, Poland; 2 Department of Pediatrics, Oncology, Hematology and Diabetology, Medical University of Lodz, Lodz, Poland

**Keywords:** Biological monitoring, Arsenic, As3MT, GST, Polymorphism, Human, Urine

## Abstract

**Purpose:**

To examine the differences in urinary arsenic metabolism patterns in men affected by occupational exposure, we performed a study on 149 participants—workers of a copper mill and 52 healthy controls without occupational exposure. To elucidate the role of genetic factors in arsenic (As) metabolism, we studied the associations of six polymorphisms: *As3MT* Met287Thr (T>C) in exon 9; *As3MT* A>G in 5′UTR; As3MT C>G in intron 6; As3MT T>G in intron 1; GSTP1 Ile105Val and GSTO2 T>C.

**Methods:**

Air samples were collected using individual samplers during work shift. Urine samples were analyzed for total arsenic and arsenic chemical forms (As^III^; As^V^, MMA, DMA, AsB) using HPLC–ICP-MS. A specific polymerase chain reaction was done for the amplification of exons and flanking regions of As3MT and GSTs.

**Results:**

The geometric mean arsenic concentrations in the air were 27.6 ± 4.9 µg/m^3^. A significant correlation (*p* < 0.05) was observed between arsenic in air and sum of iAs +MMA and iAs. *As3MT* (rs3740400) GG homozygotes showed significantly (*p* < 0.05) higher %iAs (21.8 ± 2.0) in urine than GC+CC heterozygotes (16.0 ± 2.1). A strong association between the gene variants and As species in urine was observed for *GSTO2* (rs156697) polymorphism.

**Conclusions:**

The findings of the study point out that the concentration of iAs or the sum of iAs + MMA in urine can be a reliable biological indicator of occupational exposure to arsenic. This study demonstrates that *As3MT* and/or *GSTs* genotype may influence As metabolism. Nevertheless, further studies investigating genetic polymorphism in occupational conditions are required.

## Introduction

**Fig. 1:**
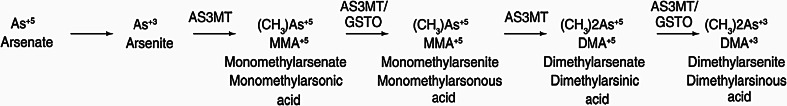
Scheme of oxidative methylation of arsenite (Reichard and Puga 2010). *As3MT* arsenic (+3 oxidation state) methyltransferase,* GSTO* glutathione S-transferase Ω

Arsenic (As) is a significant global environmental toxicant and As contamination of soil, and drinking water is a problem threatening human health all over the world. Humans are exposed to As through the intake of air, food and water and occupational exposure occurs in several industries including gold mining and smelting operations. It is well established that chronic exposure to As is associated with skin, lung and bladder cancers (IARC 1987; Helene et al. 2007; Järup et al. 1989; Lauwerys and Hoet 2001) as well as vascular diseases and hepatotoxicity (NRC 2001). The biotransformation pathway of As consists of several change in the oxidative state, oxidative methylation, producing at least four metabolites (Fig. 1). Inorganic As (iAs) is metabolized by reduction in pentavalent iAs to the trivalent form (As^III^), followed by oxidative methylation to monomethylated As (MMA), further reduction from pentavalent MMA to trivalent one and final methylation to dimethylated As (DMA) (Vahter 1999, 2002). Recently, a reductive methylation pathway has also been described (Tseng 2009). Methylated As is less toxic than the inorganic form, and methylation has been considered to be a detoxification reaction. However, recent studies have shown that methylated As^III^ is more cytotoxic and genotoxic than arsenate and arsenite (Styblo et al. 2000). Following arsenic exposure, 40–60 % of arsenic intake is eliminated through urine. It should also be mentioned that the majority of the environmentally exposed population groups studied so far have on average 10–30 % of inorganic As, 10–20 % of MMA and 60–70 % of DMA in urine, but considerable inter-individual variations have been observed, which may be a result of genetic polymorphism in the methylation capacity of arsenic (Vahter 1999).

Due to differences in toxicity of various arsenic forms, a speciation analysis is needed to distinguish between its toxic and non-toxic forms. Therefore, speciation analysis is required to clarify the health risk of arsenic intake.

Urinary levels of arsenic are generally regarded as a good measure and biomarker of exposure, although measurements of total arsenic in urine do not contain information concerning arsenic species, thereby complicating the assignment of toxicity and potential health risk of various species of As. Quantitative determination of the amount of a specific element is particularly important and that is why speciation methods are considered essential for drawing accurate conclusions in arsenic exposure and risk assessment. For many years, biological monitoring of occupational exposure to arsenic has been based on the determination of the sum of iAs and methylated metabolites DMA and MMA in urine. Over 30 arsenic species have been identified, including inorganic arsenic species (trivalent and pentavalent), methylated species, arsenosugars, arsenolipids and thio-arsenic species (Morton and Mason 2006). Although seafood has been traditionally regarded as a source of organoarsenic compounds, there is evidence of additional sources such as wild mushrooms, rice and plant species (Orloff et al. 2009). This fact may modify urinary excretion of the metabolites and thereby affect the specificity of this method. Presence of As species in the diet may lead to overestimation of occupational exposure if the workers have eaten fish and/or seafood within the 48 h prior to the testing (Airbouine and Wilson 1992).

Arsenic metabolism is regulated by several transferase genes.

Two main groups of genes have been associated with arsenic metabolism: As3MT (methyltransferases) and GSTs (glutathione S-transferases). Both oxidative and reductive metabolic pathways of arsenic (III) methyltransferase (*As3MT*), previously called Cyt19, play an important role in As methylation. The human *As3MT* gene is approximately 32-kb long and it is composed of 11 exons (Wood et al. 2006). Genetic factors are among the critical factors for As metabolism (Vahter 2000). Non-synonymous SNPs in the *As3MT* coding regions: Arg173Trp, C>T (rs35232887) in exon 6, Met287Thr, T>C (rs11191439) in exon 9 and Thr306Ile, C>T (rs34566438) in exon 10 were extensively investigated. The Met287Thr polymorphism has been found to be related to an increased percentage of MMA in urine of the Central European population (Lindberg et al. 2007) and miners in Chile (Hernandez et al. 2008). Polymorphism in the promoter regions, including 5′UTR *As3MT* (rs7085104), may affect the expression of the *As3MT* gene, which in turn is likely to affect the metabolite pattern. GST is an enzyme that detoxifies xenobiotics via a conjuction reaction with glutathione (GSH), and it has been suggested that polymorphic variants may result in having different capacities to metabolize arsenic. However, data regarding the methylation capacity of iAs or MMA, associated with these SNPs, are inconsistent (Engström et al. 2009).

The purpose of the present study was to evaluate the impact of 4 polymorphisms (rs11191439, rs7085104, rs3740393, rs3740400) in *As3MT* and two polymorphisms in GST (*GSTP1* rs 1695, *GSTO2* rs156697) on As metabolism in a Polish sub-population occupationally, but not environmentally, exposed to As. The second aim of the study was to compare validity of various biomarkers of exposure to arsenic in occupational setting and to find out whether determination of iAs or the sum of iAs and MMA in urine would be useful for the purpose of assessment of exposure to inorganic arsenic.

## Materials and methods

### Subjects and samples

This study was performed on copper mill workers (males *n* = 149) from the southwestern part of Poland with the mean age of 47.6 ± 8.7 (range 26–61). They were equipped with protective clothing as well as with half face mask respirators. Urine and blood samples were collected after their shifts. Before samples collection, the workers were asked to remove their work clothes and wash their hands. In order to ascertain data on the excretion of arsenic species in the general population, 52 men, with the mean age of 41.8 (range 28–58), were assessed as a control group. They were not occupationally exposed to As and lived in areas not polluted with arsenic from industrial plants. Each worker and control was asked to complete a questionnaire including detailed information, inter alia, on the frequency of intake of fish or other seafood. The study was approved by the Ethics Committee of the Nofer Institute of Occupational Medicine in Lodz. The characteristics of the examined population are shown in Table 1.

**Table 1:** Characteristics of the studied groups

	Workers exposed occupationally (*n* = 149)	Control group (*n* = 52)
	Mean ± SD and range/or frequency	Mean ± SD and range/or frequency
Age (years)	47.6 ± 8.7 (26–61)	42.0 ± 10.2 (28–58)
*Smoking history*		
Never	72 (48.3 %)	20 (38.5 %)
Former	20 (13.4 %)	17 (32.6 %)
Current	57 (38.3 %)	15 (28.8 %)
BMI (kg/m^2^)	28.4 ± 3.4 (20.0–42.0)	26.7 ± 3.4 (18.5–38.9)
Period of As exposure (years)	23.2 ± 10.9 (1–41)	–

### Analyses of As in the air (As-A)

Air samples were collected via the individual dosimetry method in the breathing zone of each worker continuously throughout a 6–7-h period of time in accordance with the sample collecting procedure contained in PN-Z-04008-7:^2002^. During the collection process, an individual dust measuring device was applied (Personal Air Samples, Vortex Standard and Vortex Standard 2 by Casella, and also EHA AIR-300 by Ekohigiena). It was used to collect the total dust at the flow of 2 l/min. The samples were collected on membrane filters made of cellulose nitrate (Sartorius 11304, diameter of pores—0.8 µm, filter diameter—37 mm) (Goettingen, Germany) and of fiberglass (Whatman GF/A, diameter 37 mm) (Buckinghamshire, UK). The total dust was collected by placing 1–3 filters simultaneously in the breathing zone of each study participant. In order to determine arsenic and its compounds in the air of the workplace, the method of inductively coupled plasma mass spectrometry (ICP-MS) was applied (version offered by NIOSH Manual of Analytical Methods, Fourth Edition Method 7301, Issue 1, 2003, Elements by ICP (Aqua Regia Ashing) and Method 7901, Issue 2, 1994, Arsenic Trioxide, as As).

ELAN DRC-e ICP-MS with Dynamic Reaction Cell (Perkin Elmer, SCIEX, USA) was used for the purpose of arsenic determination in dust.

### Analyses of As in urine (As-U)

Urine samples were collected from the workers as well as from the controls. Workers provided spot urine samples immediately after the shift-end on the second day after exposure. The samples were collected after the workers had removed their work clothes and washed their hands. The control subjects provided spot urine samples the morning they came to the laboratory. In all the urine samples, creatinine content was determined.

To limit the interconversion of As species, urine samples were frozen after collection and stored at <−20 °C until analysis. Prior to the dilution, the samples were centrifuged at 4,000 rpm for 10 min and next, the supernatant was diluted tenfold with 1.0 % HNO_3_ for the total arsenic and the mobile phase for the speciation analysis. ELAN DRC-e ICP-MS with a Dynamic Reaction Cell (Perkin Elmer, SCIEX, USA) was used for arsenic determination. A cyclonic spray chamber, a Meinhard nebulizer and a peristaltic four-channel pump were used. The instrument Series 200 HPLC (Perkin Elmer, SCIEX, USA) was applied to separate arsenic chemical forms. An Anion Exchange, Hamilton PRP-X100 column (4.1 mm i.d. × 250 mm × 10 µm) was used under the following conditions: 5 mM (NH_4_NO_3_) ammonium nitrate/5 mM (NH_4_H_2_PO_4_) (dibasic), flow rate 1.5 ml/min, injection volume 100 µl.

Certified reference material SRM 2669 (human urine) from the National Institute of Standard and Technology (NIST) with a certified range of (values determined by laboratory) As_tot_.44.4–57.0 µg/l (50.2 ± 5.4); As^III^ 4.72–5.34 µg/l (4.95 ± 0.71); As^V^ 5.21–7.11 µg/l (5.76 ± 0.31); MMA-6.62–7.74 µg/l (7.05 ± 0.55); DMA-24.6–26.0 µg/l (25.5 ± 1.5); AsB-1.35–1.51 µg/l (1.40 ± 0.08) were examined at the beginning and at the end of the analysis. The laboratory participates in the external quality program for the total arsenic determination organized by the Institute of Occupational Social and Environmental Medicine of the University of Erlangen, Nuremberg (G-EQUAS). To evaluate the efficiency of the first and the second step of methylation, the ratios of inorganic arsenic and metabolites were calculated.

### Analytical performance

HPLC–ICP-MS method was used for the measurement of 5 arsenic species (As^III^, As^V^, MMA, DMA and AsB) (Rabieh et al. 2008; Suzuki et al. 2009). The ICP-MS instrument was used in dynamic reaction cell mode (DRC). The limits of detection (LODs) were, respectively: As_tot._:0.026; As^III^:0.045; As^V^:0.11; MMA:0.12; DMA:0.07 and AsB:0.027 µg/l. The LOD was calculated as three times the standard deviation from the lowest concentration from the calibrate curve. All the species were separated, and all peaks have eluted in 10 min. Arsenic species were calibrated in range 1–100 µg/l.

### Creatinine determination

Creatinine was determined using colorimetric Jaffe method [Über den Niederschlag, welchen Pikrinsäre in normalem Harn erzeugt und über eine neue Reaction des Kreatinins by Max Jaffe (1886)]. Analysis was carried out at 520 nm on Lambda EZ210 spectrometer PerkinElmer (USA).

### Genotyping of polymorphisms in As3MT/GSTs

Genomic DNA was isolated from the whole blood samples using QIAamp DNA Blood Mini Kit (Qiagen). Genotyping of *As3MT* Met287Thr; T>C in exon 9 (rs7085104) (Assay ID: C_3284563_10); A>G 5′ terminal polymorphism (rs11191439) (Assay ID: C_31979150_10); As3MT C>G (rs 3740393) in intron 6(Assay ID: C_25804287_10); As3MT T>G (rs 3740400) in intron 1(Assay ID: C_27510174_10); GSTP1 Ile105Val (rs 1695) (Assay ID: C_3237198_20) and GSTO2 T>C (rs 156697) Assay ID: C_3223136_1) was conducted by real-time PCR CFX96 System (BioRad, Hercules, CA, USA) assays using pre-design 5′ nuclease allelic discrimination (TaqMan^®^) assays with Genotyping Master Mix (Applied Biosystems, Foster City, CA, USA).

### Statistical analyses

We tested the deviation from the Hardy–Weinberg equilibrium using the Chi-square test. Shapiro–Wilk test was used to determine the normality of distribution. Variables which deviated from normality underwent logarithmic transformation and were presented in the text as geometric means with geometric standard deviations. For pairwise comparisons, the Student’s *t* test was applied and for comparisons of more than two groups, the analysis of variance (ANOVA) was performed instead with Tukey’s test for post hoc comparisons. The effects of the studied polymorphisms were tested for both: polymorphic allele carriage effect and polymorphic allele homozygosity. Correlations were evaluated using the Pearson’s correlation tests. Multivariate analyses were performed using general linear regression models. The level of *p* < 0.05 was considered as statistically significant. All statistical analyses were performed using the Statistica (Statsoft, Tulsa, OK, USA) software package.

## Results

### Concentration of As in the air

The airborne arsenic concentrations (As-A), measured using individual sampling, varied from 0.2 to 275.6 µg/m^3^. The geometric mean of arsenic concentration in the air was higher than MAC (maximum admissible concentration) valid in Poland, equal 10 µg/m^3^. Concentrations above the MAC value were found in 82 cases among the 149 investigated subjects. None of the controls had elevated As-A levels. We found weak but statistically significant linear correlations between arsenic concentration in the air and urinary As^III^ (*r* = 0.334; *p* < 0.05); As^III^ + As^V^ (0.300; *p* < 0.05) and As^III^ + As^V^ + MMA (*r* = 0.286; *p* < 0.05) levels in the copper mill workers. No statistically significant correlation was found between air As concentration and As^III^ + As^V^ + MMA + DMA and As_tot_ in urine of the copper mill workers (Table 2).

**Table 2:** Relationship between inorganic arsenic concentration in the air and different forms of the biomarkers in urine

As_2_O_3_ in air (µg/m^3^) GM ± GSD	Arsenic metabolites	*N*	Regression equation	Correlation coefficient
27.6 ± 4.9	As^III^	149	*y* = 17.20 + 1.85x	*r* = 0.334*
	As^III^ + As^V^	149	*y* = 17.99 + 1.13x	*r* = 0.300*
	As^III^ + As^V^ + MMA	149	*y* = 17.24 + 0.80x	*r* = 0.286*
	As^III^ + As^V^ + MMA + DMA	149	*y* = 19.82 + 0.24x	*r* = 0.188
	As total	149	*y* = 26.30 + 0.04x	*r* = −0.09

* Statistically significant at *p* < 0.05

### Concentration of the total As (As_tot.._) and As species in urine

The levels of the total arsenic (As_tot_.), inorganic arsenic (As^III^, As^V^) and metabolites (MMA and DMA) were determined in the urine samples of 149 copper mill workers and 52 control subjects. The influence of age, BMI, time of exposure and smoking habits on As and metabolites concentration was investigated. The results of speciation analysis of arsenic in urine of the copper mill workers and in control group are presented in Table 3. In 81 cases, the concentrations of As_tot._ in urine were under the values of biological exposure index currently valid in Poland (35 µg/l). Total urinary arsenic concentrations, as well as urinary arsenic species levels in urine of the copper mill workers were significantly higher (*p* < 0.05) than the concentrations and levels observed in the control group (Table 3). In the control group, AsB was excreted most often, then DMA and MMA. Inorganic arsenic in the majority of cases was below the limit of quantification. We proved that either As_tot._ or DMA and AsB levels in urine of the workers who confirmed fish consumption about 3 days before examination were statistically higher (*p* < 0.05) than those in the workers who did not confirm fish consumption (respectively, 56.8 ± 3.1 vs. 33.0 ± 2.2; 25.7 ± 3.6 vs. 15.6 ± 2.5; 19.3 ± 4.3 vs. 3.6 ± 3.1). Similar trend of urinary As excretion depending on fish consumption was proved within the control group, but concentrations of As species were significantly lower as compared to the workers group (*p* < 0.001) (for As tot. 22.7 ± 4.2 vs. 4.0 ± 1.9 µg/l; for DMA 10.9 ± 2.8 vs. 2.7 ± 2.0 µg/l; for AsB 8.6 ± 7.9 vs. 0.6 ± 2.1 µg/l). Statistically significant differences between As_tot._ and iAs concentrations in the copper mill workers in relation to smoke addiction were observed. Smokers had significantly higher (*p* < 0.05) As_tot._ concentration as compared to the controls (46.4 ± 2.6 vs. 33.7 ± 2.2 µg/l) and significantly higher (*p* < 0.001) iAs concentration (7.6 ± 2.6 vs. 5.7 ± 3.3 µg/l).

**Table 3:** Geometric mean values of arsenic and chemical species in urine of copper mill workers and control group

Parameter	*N*	Total As		AsB		As^III^		DMA		MMA		As^V^	
GM ± GSD (range)		µg/l As	µg/g creat.	µg/l As	µg/g creat.	µg/l As	µg/g creat.	µg/l As	µg/g creat.	µg/l As	µg/g creat.	µg/l As	µg/g creat.
Copper mill workers	149	37.8 ± 2.5 (2.8–511.6)	34.8 ± 2.1 (5.2–319.6)	5.5 ± 4.2 (0.2–370.6)	5.0 ± 3.6 (0.1–241.3)	4.0 ± 3.1 (0.1–37.7)	3.6 ± 2.8 (0.1–32.3)	15.6 ± 2.7 (0.4–112.3)	14.4 ± 2.3 (1.0–85.2)	3.7 ± 2.2 (0.4–26.0)	3.4 ± 2.1 0.3–17.3)	2.0 ± 3.4 (0.1–23.8)	1.8 ± 2.9 (0.1–50.4)
Control group	52	6.7 ± 3.4 (1.2–124.2)	5.4 ± 3.3 (0.7–73.9)	1.3 ± 5.6 (0.15–86.8)	1.0 ± 5.1 (0.4–73.1)	0.3 ± 2.7 (<LOD–1.7)	0.3 ± 2.8 (<LOD–1.09)	4.1 ± 2.8 (1.0–34.8)	3.4 ± 2.5 (1.5–23.5)	0.3 ± 2.3 (0.05–1.3)	0.3 ± 2.5 (0.11–1.2)	0.2 ± 2.4 (<LOD–0.8)	0.2 ± 2.5 (<LOD–0.6)
*p*		*p* < 0.05*	*p* < 0.05	*p* < 0.05	*p* < 0.05	*p* < 0.05	*p* < 0.05	*p* < 0.05	*p* < 0.05	*p* < 0.05	*p* < 0.05	*p* < 0.05	*p* < 0.05

* Statistical significance as compared to the controls

### Haplotype analysis

**Fig. 2:**
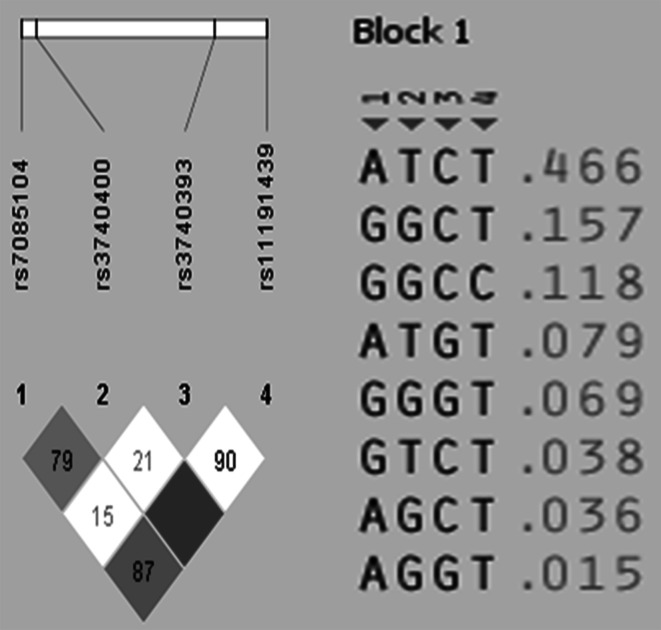
LD values for As3MT SNPs

Haplotype analysis of the four genotyped loci in *As3MT* showed that rs11191439 and rs3740393 were in high linkage disequilibrium similarl to rs3740400 and rs7085104. Therefore, only rs3740393 and rs3740400 were chosen for the adjusted multivariate analysis. The effects were the strongest for the rs3740400 genotypes and were significantly associated with higher %iAs. (Table 4; Fig. 2).

**Table 4:** Significance of the As3MT (rs3740400) and GSTO2 (rs156697) genotypes in the excretion profiles in the exposed group

*As3MT rs3740400*			*p*	*p* for polymorphic allele carriage	*p* adjusted for age, work-years	*p* for polymorphic homozygote	*p* adjusted for age, work-years
%iAs	TT *N* = 51	16.2 ± 2.02	0.085	0.567	0.586	0.028	0.097^#^
	TG *N* = 67	15.7 ± 2.1					
	GG *N* = 31	21.8 ± 2.0					
*GSTO2 rs 156697*							
%MMA	TT *N* = 67	13.5 ± 1.8	0.028	0.007	0.013	0.572	0.656^&^
	TC *N* = 70	10.1 ± 2.0					
	CC *N* = 12	10.4 ± 2.1					
MMA/iAs	TT *N* = 67	0.7 ± 2.4	0.145	0.049	0.046	0.577	0.567*
	TC *N* = 70	0.5 ± 2.2					
	CC N = 12	0.5 ± 3.2					
DMA/MMA	TT *N* = 67	3.5 ± 2.2	0.055	0.016	0.027	0.477	0.547^$^
	TC *N* = 70	4.9 ± 2.2					
	CC *N* = 12	5.0 ± 2.3					

Equations for the regression models are given below the table—all the models were built for log-transformed variables

^#^Log(iAs) = 2.485 + 0.014*Age−0.014*work duration + 0.034 (if rs3740400 G carrier)

^&^Log(%MMA) = 2.614−0.004*Age−0.001*work duration −0.044 (if rs156697 C carrier)

* Log(MMA/iAs) = 0.068−0.018*Age + 0.011*work duration −0.145 (if rs156697 C carrier)

^$^Log(DMA/MMA) = 1.478−0.003*Age + 0.004*work duration + 0.161 (if rs156697 C carrier)

### Genetic polymorphism in As3MT/GSTs and As species in urine

All polymorphisms were consistent with the frequencies expected from the Hardy–Weinberg Equilibrium (Rodriguez et al. 2009) (Table 5). In the analyzed occupational exposure group, two individuals (1.3 %) with two variant *As3MT* CC (rs11191439) alleles and four individuals (2.7 %) with two variant As3MT GG (rs3740393) alleles were recorded. The results showed significant associations between *As3MT* G>C (rs3740400) and the As concentration and metabolite pattern in the urine of the occupationally exposed workers. The workers with variant *As3MT* GG allele homozygotes were characterized by a higher percentage of iAs than CC+CG common homozygotes/heterozygotes (Table 4). On the other hand, *As3MT* Met287Thr (T>C) (rs11191439); *As3MT* A>G (rs7085104); As3MT C>G (rs3740393) polymorphisms had no significant relation to any of the indicators of the As exposure and metabolic capacity.

**Table 5:** Geometric mean concentrations of different forms of As in urine and their ratios according to SNPs of the As3MT and GST distribution in the occupationally exposed workers

SNP ID	*n*	iAs (%) (range)	DMA (%) (range)	MMA (%) (range)	DMA/MMA (range)	MMA/iAs (range)
*As3MT C*>*G rs 3740393*						
*CC*	100	16.2 ± 2.2 (2.9–53.4)	47.9 ± 1.6 (22.0–88.2)	11.1 ± 1.9 (2.2–35.3)	4.3 ± 2.4 (1.1–39.3)	0.6 ± 2.4 (0.07–8.3)
*CG*	45	18.9 ± 1.9 (3.6–70.2)	49.3 ± 1.4 (8.7–85.8)	12.8 ± 1.8 (3.5–32.6)	3.8 ± 2.2 (1.4–24.8)	0.6 ± 2.3 (0.05–3.3)
*GG*	4	17.0 ± 2.2 (5.7–31.5)	50.9 ± 1.3 (37.1–67.1)	9.4 ± 2.6 (3.9–37.1)	5.4 ± 3.3 (1.0–17.0)	0.4 ± 4.4 (0.2–3.71)
*As3MT T*>*C rs 3740400*						
*TT*	51	16.2 ± 2.02 (1.9–39.7)	49.8 ± 1.4 (22.0–74.4)	10.7 ± 1.9 (2.2–32.6)	4.6 ± 2.4 (0.5–32.8)	0.6 ± 2.5 (0.07–8.3)
*TG*	67	15.7 ± 2.1 (2.9–55.9)	48.7 ± 1.6 (4.6–88.2)	11.3 ± 2.0 (2.3–37.1)	4.3 ± 2.6 (1.0–17.6)	0.6 ± 2.4 (0.05–5.3)
*GG*	31	21.8 ± 2.0 (6.1–70.2)	45.4 ± 1.5 (8.7–70.9)	13.6 ± 1.7 (3.9–29.6)	3.3 ± 2.0(1.0–17.0)	0.6 ± 2.4 (0.2–3.9)
*GSTP1 rs 1695*						
*Ile/Val*	52	19.0 ± 1.8 (5.4–40.5)	48.5 ± 1.4 (11.4–85.8)	11.4 ± 1.8 (2.1–30.9)	4.2 ± 2.2 (0.5–32.8)	0.5 ± 2.0 (0.15–1.7)
*Ile/Ile*	81	16.4 ± 2.2 (1.9–70.2)	48.4 ± 1.6 (4.6–89.3)	11.5 ± 2.0 (2.2–37.1)	4.2 ± 2.5 (1.0–39.3)	0.6 ± 2.7 (0.05–8.3)
*Val/Val*	15	14.3 ± 2.1 (3.3–50.8)	47.7 ± 1.6 (14.7–72.8)	12.2 ± 1.7 (4.5–35.3)	3.9 ± 2.4 (0.4–10.5)	0.7 ± 2.2 (0.18–2.9)
*GSTO2 rs 156697*						
*TT*	67	17.1 ± 2.2 (1.9–70.2)	47.2 ± 1.5 (8.7–88.2)	13.5 ± 1.8 (2.1–35.3)	3.5 ± 2.2 (1.1–14.5)	0.7 ± 2.4 (0.4–8.3)
*TC*	70	16.6 ± 2.0 (1.9–53.4)	49.0 ± 1.6 (4.6–89.3)	10.1 ± 2.0 (2.2–37.1)	4.9 ± 2.5 (0.5–39.3)	0.5 ± 2.2 (0.07–3.7)
*CC*	12	19.3 ± 2.1 (6.9–55.9)	51.7 ± 1.3 (35.5–73.9)	10.4 ± 2.1 (2.2–21.1)	5.0 ± 2.3 (1.8–34.0)	0.5 ± 3.2 (0.05–1.9)

*p for HWE p* < 0.05

Significant associations were noted (Table 4) between *GSTO2* (rs156697) and the As concentration and the metabolite pattern in urine, but not for *GSTP1* (rs1695). No statistically significant differences were stated with regard to the distribution of individual alleles between the workers and the control group (Table 6).

**Table 6:** Comparison of the frequencies of As3MT in different populations

Population	*N*	Allele frequencies		
rs11191439 SNP ID 14458		T	C	
Japanese	370	0.990	0.010	Fujihara et al. (2007)
Caucasian–American	60	0.900	0.100	Wood et al. (2006)
Central European	411	0.891	0.109	Lindberg et al. (2007)
Present study workers	149	0.850	0.150	
Control group	52	0.900	0.100	

rs7085104 SNP ID 4602		A	G	
Japanese	141	0.587	0.413	Fujihara et al. (2009)
European	–	0.680	0.320	HapMap CEU (www.hapmap.org)
Argentinean	112	0.760	0.240	Engström et al. (2007)
Present study workers	149	0.580	0.420	
Control group	52	0.670	0.330	

rs3740393 SNP ID 12390		C	G	
Japanese	141	0.373	0.628	Agusa et al. (2011)
European	224	0.696	0.304	HapMap CEU (www.hapmap.org)
Argentinean	112	0.720	0.280	Fujihara et al. (2009)
Present study workers	149	0.820	0.180	
Control group	52	0.830	0.170	

rs 3740400 SNP ID 5194		T	G	
European	226	0.381	0.619	HapMap CEU (www.hapmap.org)
Present study workers	149	0.570	0.430	
Control group	52	0.660	0.340	

## Discussion

Carrying out biological monitoring for assessment of exposure to inorganic arsenic both in occupational as well as environmental exposure is based mostly on the assessment of concentrations of arsenic and its metabolites in urine as biomarkers of exposure. In the past, the tool for biological monitoring of exposure to iAs was performance of assessment based on determinations of the total arsenic. It was due to the lack of suitable analytical techniques allowing for separation of individual chemical forms of As. That kind of biomonitoring made it possible to assess exposure from all sources, simultaneously favouring overestimation of exposure due to the presence of organic forms (AsB, AsCh) coming mainly from the food, and it increased the concentration sometimes to even more than 200 µg/l. Currently, biological monitoring of occupational exposure assessment is based on determination of the sum of inorganic arsenic concentrations and main methylated metabolites, MMA acid and DMA in urine. In the carried out studies aiming at verification of the usefulness of determination of the sum of iAs +MMA + DMA as a biomarker of exposure, a linear, statistically significant correlation between inorganic arsenic concentrations in the air and concentrations of inorganic arsenic and the sum of iAs and MMA in urine in the samples collected at the end of a work shift was obtained. Similar correlations have been presented by others authors involved in the assessment of occupational exposure to iAs (Hakala and Pyy 1995; Offergelt et al. 1992). A statistically significant correlation between iAs concentrations in the air and the currently applied biomarker (iAs + MMA + DMA) was not obtained. It is probably a result of the increased concentrations of DMA acid, especially in the individuals who confirmed the intake of fish meals before the study. Content of DMA acid in the food of sea origin was confirmed in scientific reports; however, an increased content of this compound may be also a result of catabolic transformations of other organic compounds of arsenic such as arsenosugars or arsenolipids coming from other types of food but having influence on DMA concentrations (Airbouine and Wilson 1992; Soleo et al. 2008; Navas-Acien et al. 2011). In the case of the individuals who declared an increased intake of fish and seafood, statistically significant differences were stated not only with regard to concentrations of As_tot_ and AsB, but also in DMA concentrations in comparison with the individuals who did not eat fish and seafood (25.7 do 15.6 µg/l, *p* < 0.05). Similar results have been achieved by Navas-Acien et al. (2011). Since fish and seafood, as Navas-Ancien et al. claim, are the main reason for a high concentration of As in urine, the authors propose to use As_tot_ minus AsB as a marker of exposure to inorganic arsenic. Other authors, among others Hata et al. (2007), proposed to consider the sum of concentrations of iAs + MMA with exclusion of DMA as a marker of exposure to arsenic. Such a form of biomonitoring was also proposed by other authors (Hakala and Pyy 1995; Offergelt et al. 1992; Apostoli et al. 1999), suggesting the values of admissible concentration of inorganic arsenic in urine (6.7, 6.2 and 5 µg/l) and referring them to a maximum admissible concentration of iAs in the air of the workplace as proposed by ACGIH—10 µg/m^3^ (current threshold limit value set by the American Conference of Governmental and Industrial Hygienists) (ACGIH 2014). Based on the carried out studies and obtained correlations (Table 2) between concentrations of iAs in the air and individual biomarkers in urine, the values of concentrations of iAs and the sum of iAs + MMA were calculated and proposed as a reference to the currently applying admissible value in the air of the workplace (TLW-TLV–10 µg/m^3^). For iAs concentrations in urine, the obtained value is 8.2 µg/l and it is similar to the values proposed by others authors (Hakala and Pyy 1995; Offergelt et al. 1992; Apostoli et al. 1999), while in the case of the sum of iAs + MMA, this value equals 12.7 µg/l. As it was shown in the control group, DMA and AsB constituted the majority of speciation forms of arsenic, then there was MMA. Inorganic forms constituted only a minor percent (Table 3). Similar distribution of concentrations of chemical forms of arsenic was obtained for the not exposed populations (Navas-Acien et al. 2011; Morton and Mason 2006; Caldwell et al. 2009). Therefore, recognition of concentrations of iAs or the sum of concentrations of iAs + MMA as biomarkers of assessment of occupational exposure to arsenic seems to be justified. On the other hand, a clear influence of individual differences in the methylation of arsenic, associated with genetic polymorphisms of metabolising enzymes, requires attention to this aspect of the next to the biomarkers of exposure.

Methylation of inorganic As to MMA and DMA is an important reaction in As biotransformation. Individual variations in inorganic As metabolism may influence the toxic effects. The most important role in arsenic metabolism in humans is played by As3MT and GSTs, and its genetic polymorphism may be associated with the susceptibility to inorganic arsenic toxicity. Results of *As3MT* genotype-dependent differences in As metabolism for most SNPs in As3MT were obtained in several studies involving individuals exposed environmentally to high As levels in water, especially from Asia and South America (Agusa et al. 2009, 2011; Engström et al. 2007, 2011; Fujihara et al. 2007, 2009; Hernandez et al. 2008; Meza et al. 2005). In the case of GSTs, the number of reports is smaller and they primarily refer to the populations of Asia and South America (Agusa et al. 2010; Caceres et al. 2010; Marcos et al. 2006; De Chaudhuri et al. 2008).

In the present study, we investigated the influence of four genetic polymorphisms of *As3MT* and two polymorphisms of GSTs that may be involved in As metabolism in occupationally exposed workers from the southwestern part of Poland. It was observed that *As3MT* polymorphism (rs 11191439, SNP ID 14458) in exon 9 and As3MT (rs 3740393, SNP ID 12390) in intron 6 may have an impact on the As methylation capacity in individuals chronically exposed to arsenic in drinking water (Agusa et al. 2009; Engström et al. 2007; Hernandez et al. 2008). The role of *As3MT* 5′UTR polymorphism (rs7085104, SNP ID 4602) has been studied in few works; however, the same location in the promoter region suggests a significant effect on the As methylation capacity (Agusa et al. 2011). As3MT polymorphism (rs 3740400, SNP ID 5194) in intron 1 was tested only by Engström et al. (2011) in the populations of Argentina and Bangladesh. The genotype frequencies for *As3MT* Met287Thr, T>C and *As3M*T 5′UTR are consistent with the results of other authors as regards the European population (Meza et al. 2005; Lindberg et al. 2007). Moreover, the frequency of C alleles in the Polish group was higher than that in the Central European population (0.15 vs. 0.11), and two cases of *As3MT* Thr287Thr (rs11191439) individuals were observed. Allele frequencies in the case of *As3MT* 5194 (rs3740400) polymorphism, and in the case of As3MT12390 polymorphism, are close to that described for European population (Table 6).

An in vitro study indicated that the *As3MT* Thr (C) variant demonstrates increased levels of enzyme activity compared to the Met (T) one and a higher capacity of increased arsenic methylation (Wood et al. 2006). In addition, studies on individuals environmentally exposed to high (Asians, South Americans) or low (Central Europeans) As levels showed that among rare C allele carriers, the first methylation capacity of As, expressed as the DMA/MMA ratio in urine, was higher than among frequent T allele carriers (Agusa et al. 2011). However, our study did not confirm these findings. We found a higher %MMA in association with the Thr allele in Met287Thr (rs 11191439). CC+TC wild-type genotype carriers had higher %MMA and MMA/iAs (first methylation step) than TT homozygotes. However, it should be emphasized that this is due to the influence of the rare allele, because these were CC homozygotes that determined the increased excretion of MMA These results are consistent with those noted by Hernandez et al. (2008) for Chile copper smelting plant workers, Agusa et al. (2009) for Vietnam residents and Lindberg et al. (2007) for the European population exposed to low As levels. Interestingly, a recent in vitro study did not confirm the differences in the methylation capacity of the *As3MT* C and *As3MT* T variants. The only difference in the DMA/MMA ratio between frequent and rare *As3MT* alleles was observed for a high As level and the presence of glutathione (GSH) in the reaction medium. The author suggests that individual variations in the GSH metabolism should also be analyzed among the modifiers of As metabolism in humans (Ding et al. 2012).

Several studies on polymorphism of *As3MT* (rs7085104) are interesting, but there is a limited number of them. The results concerning the genotype association of *As3MT* 4602 (rs7085104) with As metabolism were not consistent among different country groups. Among the subjects, *As3MT* 4602 GG carriers had lower %DMA and higher %iAs as well as MMA compared to the other genotypes. Although there were no significant differences, the results are consistent with the data presented by Agusa et al. 2009. Contrary to these results, Valenzuela et al. (2009) noted a decrease in %MMA with GG homozygotes in the Mexican population. The effect of *As3MT* polymorphism (rs 7085104) was also studied by Schläwicke Engström et al. (2009) in an Argentinean female population. He noted an influence of this polymorphism on %MMA and %DMA, but a much stronger relationship was manifested by rs 3740400 polymorphism. This is consistent with the results obtained in the present work. This polymorphism showed a statistically significant effect on the profile of the excreted metabolites (Table 4). Recessive homozygotes excreted an increased percentage of inorganic arsenic in comparison with the other genotypes. Furthermore, only this polymorphism in the haplotype analysis shows a functional effect, which has also been suggested by Engström et al. (2007). In the case of As3MT rs12390 polymorphism, interesting relationships are observed. Meza et al. (2005) pointed to a higher DMA/MMA ratio in CG+CC than in GG homozygotes, but only in children, and Chung et al. (2009) showed a reduced percentage of MMA excretion in the case of the GC genotype, compared to the GG genotype in the Taiwanese population. In the studied population, no such relationship was found. Apart from methyltransferase, an important group of enzymes involved in the metabolism of arsenic are glutathione transferases. In the present work, we investigated the impact of polymorphism within *GSTO2* T>C (rs156697) and *GSTP1* Ile105Val (rs1695). The results presented by Marcos et al. (2006), Agusa et al. (2010), Paiva et al. (2010), and De Chaudhuri et al. (2008) indicate the possible impact of polymorphism within these genes on the differences in metabolism and susceptibility to arsenic influence in population groups exposed to it environmentally and occupationally. In the case of research conducted by Marcos in 105 employees working at a copper smelter, the results are consistent with these obtained in the current study. Val/Val homozygotes tend to excrete smaller amounts of DMA than other genotypes, but the differences are not statistically significant (*p* = 0.0615). More attention has been paid to the effect of polymorphism concerning the omega class glutathione S-transferases, which is probably connected with the antioxidative action of these enzymes. *GSTO2*, non-synonymous Asn142Asp (rs156697) in the European population show important influence of this polymorphism and the possible varying effects on the toxic impact of arsenic. There are large differences in the frequencies of particular genotypes in specific populations and they mainly are related to the Asp/Asp genotype. Paiva et al. (2010) showed an increased percentage of excreted DMA in patients with the variant allele, indicating a possible protective role of this genotype. It is in fact the second degree of the methylation process responsible for creating the final, least toxic metabolite. In other works, such dependence has not been found (De Chaudhuri et al. 2008; Agusa et al. 2010; Xu et al. 2009). In contrast to the above-mentioned authors, in the current work, for the occupationally exposed population, statistically significant relationships were noted (Table 4) for this polymorphism in the dominant model. TT homozygotes (Asn/Asn) excrete an increased percentage of MMA, and the MMA/iAs and DMA/MMA ratios are also significantly different compared to the other genotypes.

Our results are in opposition to those obtained in other studies covering other populations and focusing on both the genetic and environmental factors. The concentration of As in the air was discussed as a factor in the methylation capacity. A methylation threshold hypothesis for iAs has been proposed, stating that occupational exposure to As at the concentrations above 300 µg/m^3^ may cause inhibition of the methylation process, and when exposure to iAs reaches a certain level or threshold, the methylation capacity begins to decline (Offergelt et al. 1992). The results of the epidemiological and experimental human studies do not support the methylation threshold hypothesis. The dose level of arsenic has little influence on the methylation efficiency (Hopenhayn-Rich et al. 1993; Vahter 2000). In the present work, the mean values of arsenic species detected in the samples were in agreement with the standard arsenic values (Table 5) (Vahter 2002), and the geometric mean concentrations of arsenic in the air were indeed much lower than the presumed level of inhibition of methylation (As-A−27.6 µg/m^3^). The hypothesis concerning the threshold of the methylation saturation is not completely explicit. There are studies demonstrating the percentage decrease in the concentrations of DMA with a simultaneous increase in the concentrations of MMA in the case of growing exposure, suggesting inhibition of the methylation process associated with raised concentrations of inorganic arsenic in the tissues. In the other side are studies with opposite results confirming higher excretion of DMA following the increased exposure (Del Razo et al. 1997). In the context of the above-mentioned hypotheses, the test results obtained during the implementation of the current project are similar to the assumptions of the methylation saturation process. Nonetheless, the results were in part consistent with other results for the general population and splicing variants of the *As3MT* and GST gene, and their function should be further investigated.

## Conclusions

The findings of the study point out that the concentration of iAs or the sum of iAs + MMA in urine can be a reliable biological indicator of occupational exposure to arsenic. The present study found that rs3740400 in *As3MT* and rs156697 in *GSTO2* were strongly associated with the %iAs, %MMA and MMA/iAs andDMA/MMA ratios in urine of the occupationally exposed workers and they may influence methylation capacity. However, further studies investigating genetic polymorphism in occupational conditions are required.
